# Retail Milk Monitoring of Influenza A(H5N1) in Dairy Cattle, United States, 2024–2025

**DOI:** 10.3201/eid3202.251332

**Published:** 2026-02

**Authors:** Natalie N. Tarbuck, John Franks, Jeremy C. Jones, Ahmed Kandeil, Jennifer DeBeauchamp, Lance Miller, Thomas Fabrizio, Karlie Woodard, Hannah J. Cochran, Madison C. Owsiany, Bryant M. Foreman, James F. Lowe, Richard J. Webby, Andrew S. Bowman

**Affiliations:** The Ohio State University College of Veterinary Medicine, Columbus, Ohio, USA (N.N. Tarbuck, H.J. Cochran, M.C. Owsiany, B.M. Foreman, A.S. Bowman); St. Jude Children's Research Hospital, Memphis, Tennessee, USA (J. Franks, J.C. Jones, A. Kandeil, J. DeBeauchamp, L. Miller, T. Fabrizio, K. Woodard, R.J. Webby); University of Illinois College of Veterinary Medicine, Urbana, Illinois, USA (J.F. Lowe)

**Keywords:** influenza, zoonoses, retail milk, dairy cows, influenza A(H5N1), federal orders, United States

## Abstract

US retail milk monitoring during April 13–May 3, 2024, identified influenza A(H5N1) viral RNA in 36% of retail milk samples, indicating widespread undetected infections in US dairy cows. After federal initiatives, reported infections more closely aligned with findings in retail milk during December 27, 2024–January 29, 2025, reflecting improved detection and control.

The emergence of influenza A virus (IAV) (H5N1) in dairy cows, first reported in March 2024, was unprecedented. Because dairy cows were not considered a typical host for IAV, initial identification was delayed after reports of nonspecific illness in lactating cattle in Texas, USA. After that initial detection, the virus was soon identified in dairy cows in several other US states, likely enabled by cattle movements (*1*–*3*). Considering the threat H5N1 posed to dairy cows, other livestock operations, and public health, the US Department of Agriculture issued federal orders requiring mandatory testing before interstate movement of animals and implementing a national testing strategy of raw, unpasteurized milk to monitor and control the outbreak. Given that H5N1 viral RNA has been detected in retail milk products (*4*), we used retail milk monitoring in this study to assess whether federal control measures are working to identify and mitigate influenza A(H5N1) in dairy herds.

## The Study

We conducted retail milk surveillance at 2 key timepoints in the influenza A(H5N1) outbreak in dairy cows. During April 13–May 3, 2024, we purchased 168 unique pasteurized milk samples from retail stores in 13 US states. During December 27, 2024–January 29, 2025, we expanded our surveillance to include 477 (469 pasteurized and 8 raw) unique milk samples purchased from retail stores in 25 US states. To better assess the geographic extent of the outbreak, we included a mix of states with confirmed H5N1 virus–infected dairy herds and states without known infected dairy herds. To reduce duplication of samples tested, we selected unique milk samples by choosing different milk plant codes and expiration dates. We identified states with IAV viral nucleic acid–positive retail milk on the basis of the location of the milk plant, rather than where the milk was purchased.

We screened retail milk samples for the presence of influenza A viral nucleic acid using real-time quantitative PCR. We extracted RNA from retail milk using the MagMAX viral/pathogen II nucleic acid isolation kit (Thermo Fisher Scientific, https://www.thermofisher.com) or the QIAGEN RNeasy Mini Kit (QIAGEN, https://www.qiagen.com), according to manufacturer instructions. We quantified the noninfectious viral load using the VetMAX-Gold SIV Detection Kit (Thermo Fisher Scientific), which was applied to bovine milk samples outside its US Department of Agriculture license. We shipped a separate aliquot of retail milk that had never been freeze-thawed to St. Jude Children’s Research Hospital (Memphis, TN, USA) for confirmation of subtype and virus viability experiments (Appendix). We ran each sample in triplicate (influenza H5b) with primers and probe sequences designed by the Centers for Disease Control and Prevention and acquired through the International Reagent Resource (https://www.internationalreagentresource.org). Reactions were performed on the ABI 7500 FAST Real-Time PCR system (Thermo Fisher Scientific).

On April 12, 2024, only 29 infected dairy herds had been reported (*3*), although we hypothesized the outbreak was more widespread. Despite the relatively small number of infected dairy herds reported, we detected IAV in 36.3% (61/168) of pasteurized retail milk samples during April 13–May 3, 2024 (Table), including in 5 states (AR, IN, MN, MO, OK) where no outbreak in dairy cows was reported at the time sampling was initiated (Figure). We found no evidence of viable virus in IAV-positive retail milk samples, as determined by in vitro cell passaging and mouse inoculation with pasteurized milk (Appendix Figure), consistent with findings that pasteurization is effective (*4*,*5*). During December 27, 2024–January 29, 2025, we expanded our surveillance but detected IAV in only 6.9% (33/477) of retail milk samples collected, all of which were processed in California (Table). We confirmed nearly all (96.8%; 91/94) of the IAV-positive retail milk samples in this study as influenza A(H5); the remaining samples were likely untyped because of limited viral RNA or sensitivity of H5 assay.

**Table:** Estimated influenza A virus prevalence in retail milk by state and study period in study of retail milk monitoring of influenza A(H5N1) in dairy cattle, United States, 2024–2025*

State	No. positive/no. tested (%)	
	Period 1	Period 2
Arizona	0/5	0/15
Arkansas	3/3 (100)	0/5
California	0/3	33/55 (60)
Colorado	5/20 (25)	0/23
Connecticut		0/2
Florida		0/24
Georgia	0/1	0/3
Idaho		0/7
Illinois		0/21
Indiana	2/21 (9.5)	0/31
Iowa		0/34
Kansas	12/15 (80)	0/5
Kentucky	0/1	0/6
Maryland	0/1	0/1
Massachusetts		0/2
Michigan	5/16 (31.3)	0/28
Minnesota	1/7 (14.3)	0/25
Missouri	1/5 (20)	0/24
Nebraska	0/4	0/9
New Jersey		0/5
New York	0/10	0/23
North Carolina		0/5
Ohio	2/18 (11.1)	0/25
Oklahoma	3/3 (100)	0/12
Pennsylvania		0/15
South Dakota		0/2
Tennessee		0/4
Texas	27/33 (81.8)	0/21
Utah		0/20
Virginia	0/2	0/13
Wisconsin		0/12
Total	61/168 (36.3)	33/477 (6.9)

*The number of states included in surveillance increased from period 1 to period 2. Period 1, March 25–May 3, 2024; period 2, December 9, 2024–January 29, 2025.

**Figure:**
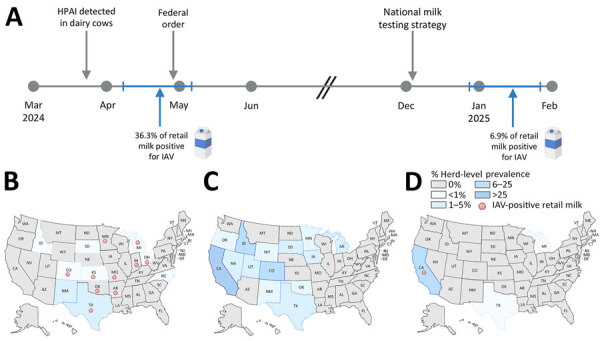
Timeline and geographic distribution of influenza A(H5N1) outbreaks in dairy cattle herds in study of retail milk monitoring of influenza A(H5N1) in dairy cattle, United States, 2024–2025. A) Timeline of detections and federal interventions. B–D) Locations of confirmed H5N1 outbreaks in dairy herds, standardized as the prevalence of infected herds relative to the total number of herds per state, on the basis of 2022 US Department of Agriculture (USDA) Census of Agriculture data (*6*). A) USDA reported outbreaks (n = 36), March 25–May 3, 2024; B) USDA reported outbreaks (n = 701), May 4–December 8, 2024); C) USDA reported outbreaks (n = 218), December 9, 2024–January 29, 2025. The red virion marks the state of processing plants where influenza A virus–positive retail milk was identified. Maps were generated using BioRender (https://BioRender.com). HPAI, highly pathogenic avian influenza; IAV, influenza A virus.

## Conclusions

The US dairy industry consists of 9.3 million cows that produce >226 billion pounds of milk annually (*6*). Shortly after the first report of influenza A(H5N1) in dairy cows, <0.1% of US dairy herds were reported as H5N1-positive, yet we detected IAV nucleic acid in 36% of retail milk samples (Figure). Our study revealed that early in the outbreak, the influenza A(H5N1) virus was more widespread than reported; the prevalence of IAV-positive retail milk was markedly higher than that of infected herds reported. Given the size of the US dairy industry, the high prevalence and low cycle threshold values detected in retail milk during April 13–May 3, 2024, suggest that a substantial number of infected cows were actively shedding virus into the milk supply, and many infections were going undetected because of limited surveillance. Those undetected cases are further supported by phylogenetic analyses that indicate that a single spillover event of influenza A(H5N1) from wild birds to dairy cows likely occurred in late 2023 and went undetected for several months (*2*), enabling opportunities for cattle movement and widespread transmission. Because initial infections were identified through passive surveillance and testing of clinically affected animals, the true extent of spread was underestimated. Those findings emphasize the importance of active surveillance and federal orders that expanded testing and reporting of influenza A(H5N1) in livestock and milk.

After the federal order on April 24, 2024, mandating premovement testing of lactating dairy cattle before interstate movement, and the subsequent implementation of the National Milk Testing Strategy on December 6, 2024 (*7*), the number of reported infected herds has grown tremendously, which is expected with increased surveillance efforts. To date, >1,000 infected herds have been reported across the United States (*3*), most of which were concentrated in California. Of note, 225 infected herds were reported in California in December (*3*), just before our second study period **(**December 27, 2024–January 29, 2025) (Figure). That report aligns more closely with patterns in our retail milk surveillance, which found IAV-positive retail milk only in California, underscoring the effectiveness of the federal orders. Those surveillance efforts not only appeared to improve the identification of infected herds but also demonstrated more limited distribution. Taken together, our findings suggest that early in the outbreak, cases in US dairy herds were widespread and went undetected, but federal regulations have since improved detection and worked to control the spread of H5N1 virus in dairy herds. Further, the role of natural immunity from prior infections must also be considered as a factor limiting transmission, yet the duration of such immunity and its ability to prevent reinfection remain unclear. Of note, despite those combined measures, the virus has not been eliminated from dairy herds (*3*).

Retail milk testing is an imperfect solution to gaps in surveillance (*4*,*8*). Because retail milk represents a composite sample derived from multiple cows and processed in bulk, it limits the ability to identify the source of H5N1 virus–infected cows and pinpoint more granular viral evolution. In addition, the location of milk processing plants provides limited geographic resolution, because milk might be transported across state lines after collection from farms before processing. Active surveillance programs are critical in cattle and other livestock, wild birds, and humans at the frontline of exposure. Evidence of 2 additional spillover events of the H5N1D1.1 genotype, identified through the National Milk Testing Strategy (*9*,*10*), highlights the complexity and uncertainty in current transmission pathways. However, those spillover events also highlight the importance of current surveillance strategies in place to identify infected herds and new evolutionary trajectories.

Appendix Additional information about retail milk monitoring of influenza A(H5N1) in dairy cattle, United States, 2024–25
