# Supplementary material for: Retail Milk Monitoring of Influenza A(H5N1) in Dairy Cattle, United States, 2024–2025
Source: Emerg Infect Dis. 2026 Feb;32(2):238–41. doi: 10.3201/eid3202.251332 (PMC12928245; doi:10.3201/eid3202.251332)
Supplement: Appendix — Additional information about retail milk monitoring of influenza A(H5N1) in dairy cattle, United States, 2024–25 [file 25-1332-Techapp-s1.pdf]

Article DOI: https://doi.org/10.3201/eid3202.251332

EID cannot ensure accessibility for supplementary materials supplied by authors. Readers who have difficulty accessing supplementary content should contact the authors for assistance.

Retail Milk Monitoring of Influenza A(H5N1) in Dairy Cattle, United States, 2024–2025

Appendix

Methods

Virus isolation from A(H5N1) rRT-PCR positive milk samples

To determine virus viability in pasteurized milk samples obtained from retail stores, virus isolation was attempted on 61 A(H5) positive samples from the first study period using tissue culture and egg propagation methods. Madin-Darby Canine Kidney cells (MDCKs, ATCC, P35) were plated at 5×10^5 cells/well in 12-well plates in growth medium (MEM, 5% fetal bovine sera, 1x penicillin/streptomycin/amphotericin B, 1 mM L-glutamine) overnight, resulting in confluent monolayers. MDCKs (n = 2 wells/milk sample) were washed 3x with sterile phosphate buffered saline (PBS) and inoculated with 1 mL milk sample prepared neat (undiluted) for 1 hr at 37°C in infection medium (MEM, 1% bovine serum albumin, 1x penicillin/streptomycin/amphotericin B, 1mM L-glutamine). Negative control wells included 1 mL infection medium only, and positive control wells were inoculated with A/bovine/Texas/98638/2024 (H5N1, A/bovine/TX) (≈ MOI 0.01). Inocula were removed, the monolayers were washed 3x with PBS, and the supernatants were replaced with 1mL infection medium. Cells were incubated for 72 hr, 37°C. Every 24 hr, monolayers were observed for influenza-induced cytopathic effect (CPE). Supernatants were collected at 72 hours post-inoculation (hpi) and assessed for hemagglutination activity (HA assay) as an indicator of virus particle presence, using chicken red blood cells (cRBCs).

Virus isolation was also attempted in 10-day old embryonated chicken eggs. Eggs (n = 3/milk sample) were inoculated with 200 µL milk diluted 1:1 in egg injection antibiotics (2×10^5

U/mL penicillin G potassium salt, 40,000 U/mL streptomycin, 20,000 U/mL Polymixin B, 4 mg/mL gentamicin in PBS). Eggs were candled daily for 72 hr to assess viability. At 72 hpi, allantoic fluid was harvested from each egg.

Both cell supernatants and egg allantoic fluid from the initial round of isolation (Passage 1, P1) were subjected to a second-round blind passage. Supernatants or allantoic fluid that were HA-negative after P1 were pooled (n = 3 samples/pool) and inoculated to MDCKs or injected into eggs as described above. Both cell and egg viability were monitored every 24 hr and HA activity was assessed at 72 hpi.

Inoculation of mice with retail milk samples

Mice at 6 weeks of age (BALB/c (Jackson Labs, median weight 17.6g)) were inoculated with 6 retail milk samples (n = 3 mice/sample) representing diversity in region of sale, milk distributor, and low Ct value (23.7 to 28.0) (Appendix Figure, Appendix Table). Once again, all samples used as inoculum were collected during the first study period. Mice were lightly anesthetized with 4% isoflurane and inoculated with 30 µL undiluted milk intranasally (IN). Positive control mice were inoculated with 10^6 $TCID_{50}$ units of A/bovine/Ohio/ B24OSU-439/2024 (H5N1, A/bovine/OH). Weight and clinical disease (disheveled coats, lethargy, anorexia, and/or neurologic involvement characterized by tremors/dystonia/limb paralysis) were assessed daily. At 14 days post-inoculation (dpi), sera was collected from the mice and tested for antibodies via hemagglutination inhibition assay (HAI) as described previously (*1*). Mice were re-challenged with 2×10^5 $TCID_{50}$ A/bovine/OH and monitored for weight loss and mortality for an additional 5 days.

Results

Virus Isolation

Virus isolation was performed on 61 retail pasteurized milk samples collected during the first study period to determine virus viability. No cytopathic effect (CPE) was observed from cells inoculated with any milk sample or negative control medium at any time point, and no HA activity was detected at 72 hpi. In contrast, our positive control, A/bovine/TX inoculated cells exhibited marked CPE ≥48 hpi, and supernatants had HA activity of 64 HA units/50 µL. Allantoic fluid from 72 hpi milk-inoculated eggs was also negative for HA activity 72 hpi.

Pooled samples (n = 2 or 3 HA negative samples per pool) were subjected to a second round of passage in both cells and eggs for 72 hpi and were also negative for CPE and HA activity, respectively.

Inoculation of Mice with Milk Samples

All mice intranasally inoculated with rRT-PCR positive (Ct 23.7 to 28.0) milk from the first study period showed no signs of clinical disease or mortality. A transient decrease in weight (≤7% starting weight) was observed in 3 mice (0077, 0085, 0060) 1 dpi after milk inoculation, but quickly resolved 24 hours later and all mice continued to gain weight until the end of the study. In contrast, A/bovine/OH virus-inoculated mice (positive controls) exhibited a gradual decrease in bodyweight and observable clinical illness as early as 1 dpi; all animals succumbed to infection 4 – 6 dpi (Appendix Figure).

To understand if sufficient antigen was present in milk samples to generate an immune response, surviving animals' sera were subjected to HAI assay 14 dpi. No animals exhibited seroconversion to the A/bovine/Texas as defined by presence of HA binding antibodies in the HAI assay. This was also recently reported by Eisfeld et al. among surviving animals inoculated with 3.3×10^3 plaque forming units of A/bovine/Texas contaminated milk (*2*). Early oral influenza vaccines containing purified protein produced detectable secretory IgA, but serum (IgG) antibodies that are the basis for clinical influenza immunity measurements were less abundant or absent (*3*). Large amount of inactivated antigen in a prime-boost scenario (*4*) as well as viral vectors capable of producing a steady amount of antigen can partially overcome these hurdles (*5*). The amount of immuno-dominant HA protein in the retail milk inoculums is unknown, and its stability, folding, and epitope continuity may also be adversely impacted by the pasteurization process. To understand if non-HAI immune correlates were present after retail milk inoculation, animals were re-challenged with a lethal dose of A/bovine/Ohio. All animals exhibited rapid weight loss beginning 3 dpi and succumbed to infection 4 – 5 dpi.

References

1. World Health Organization. WHO manual on animal influenza diagnosis and surveillance. 2002 [cited 2024 May 30]; https://iris.who.int/handle/10665/68026

2. Eisfeld AJ, Biswas A, Guan L, Gu C, Maemura T, Trifkovic S, et al. Pathogenicity and transmissibility of bovine H5N1 influenza virus. Nature. 2024;633:426–32. PubMed https://doi.org/10.1038/s41586-024-07766-6

3. Avtushenko SS, Sorokin EM, Zoschenkova NY, Zacharova NG, Naichin AN. Clinical and immunological characteristics of the emulsion form of inactivated influenza vaccine delivered by oral immunization. J Biotechnol. 1996;44:21–8. PubMed https://doi.org/10.1016/0168-1656(95)00105-0

4. Quan FS, Compans RW, Kang SM. Oral vaccination with inactivated influenza vaccine induces cross-protective immunity. Vaccine. 2012;30:180–8. PubMed https://doi.org/10.1016/j.vaccine.2011.11.028

5. Braun MR, Flitter BA, Sun W, Tucker SN. An easy pill to swallow: oral recombinant vaccines for the 21st century. Curr Opin Immunol. 2023;84:102374. PubMed https://doi.org/10.1016/j.coi.2023.102374

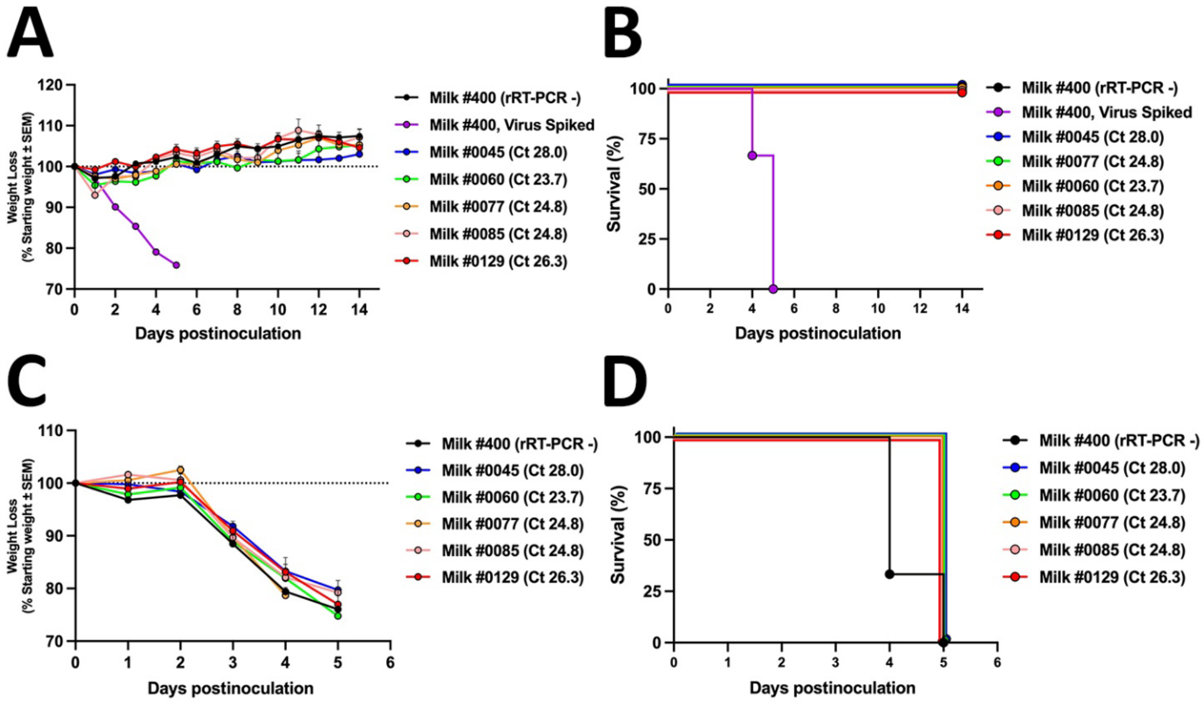

Appendix Figure. Inoculation of mice with milk samples. Mice (n = 3 per group) received A, B) an intranasal inoculum (30 μL, IN) of undiluted rRT-PCR positive retail milk (Ct value indicated), rRT-PCR negative milk, or negative milk spiked with 10^6 $TCID_{50}$ units A/bovine/OH/B24OSU-439/2024. Surviving animals received C, D) a secondary challenge of 5 x10^2 $TCID_{50}$ units of A/bovine/OH/B24OSU-439/2024 14 days later. A,C) Morbidity and B,D) mortality were monitored at the indicated timepoints.
